# Evaluation and Management of Proximal Humerus Fractures

**DOI:** 10.1155/2012/861598

**Published:** 2012-12-18

**Authors:** Ekaterina Khmelnitskaya, Lauren E. Lamont, Samuel A. Taylor, Dean G. Lorich, David M. Dines, Joshua S. Dines

**Affiliations:** Sports Medicine and Shoulder Service, Orthopaedic Trauma Service, Hospital for Special Surgery, 535 East 70th Street, New York, NY 10021, USA

## Abstract

Proximal humerus fractures are common injuries, especially among older osteoporotic women. Restoration of function requires a thorough understanding of the neurovascular, musculotendinous, and bony anatomy. This paper addresses the relevant anatomy and highlights various management options, including indication for arthroplasty. In the vast majority of cases, proximal humerus fractures may be treated nonoperatively. In the case of displaced fractures, when surgical intervention may be pursued, numerous constructs have been investigated. Of these, the proximal humerus locking plate is the most widely used. Arthroplasty is generally reserved for comminuted 4-part fractures, head-split fractures, or fractures with significant underlying arthritic changes. Reverse total shoulder arthroplasty is reserved for patients with a deficient rotator cuff, or highly comminuted tuberosities.

## 1. Introduction

Proximal humerus fractures are commonly encountered fractures in general orthopaedic practices. Treatment should focus on maximizing a patient's functional outcome and minimizing pain. Understanding the functional anatomy of the proximal humerus as it relates to fracture is paramount to achieving these goals. Intervention options range from nonoperative modalities to osteosynthesis, and in select cases arthroplasty. This paper will review relevant anatomy, common fixation constructs, appropriate indications for prosthetic replacement, and the authors' preferred treatment algorithm.

## 2. Anatomy

The glenohumeral joint is the most mobile joint in the body, resulting from a series of complex interactions among bone, muscle, and soft tissue forces. An appreciation for this anatomy enables the surgeon to effectively restore function in the setting of fracture.

The proximal humerus includes the humeral head, greater tuberosity, lesser tuberosity, and the humeral shaft. In the sagittal plane, the humeral head is retroverted an average of 30 degrees relative to the humeral shaft [1]. In the coronal plane, it is angled 130 to 150 degrees cephalad relative to the diaphysis. Fractures through the anatomic neck can result in significant vascular compromise to humeral head leading to avascular necrosis [2].

In neutral rotation, the greater tuberosity forms the lateral border of the proximal humerus. The lesser tuberosity, which sits directly anterior in this position, becomes profiled medially when the humerus is internally rotated—this creates a rounded silhouette “lightbulb sign” on radiograph. The long head of the biceps passes between the two tuberosities in the intertubercular groove, approximately 1 cm lateral to the midline of the humerus, and its relationship is an important landmark during fracture reduction [2].

**Figure 1:**
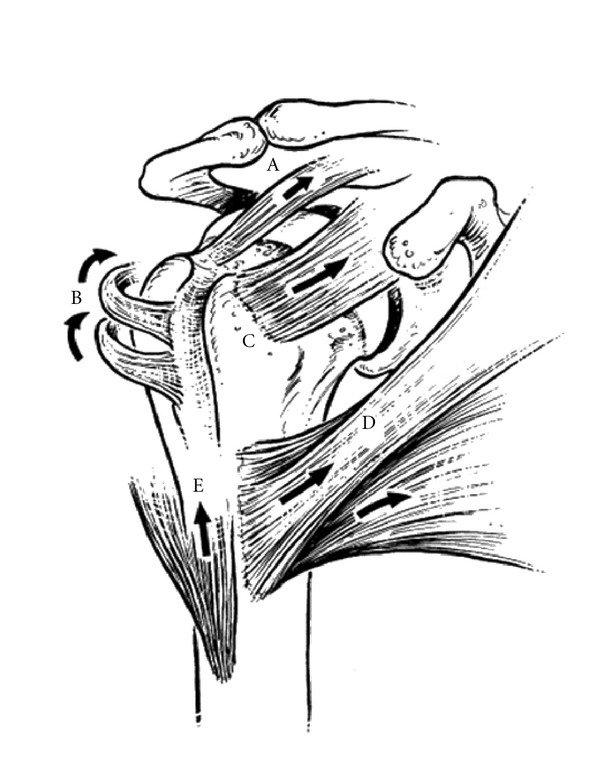
This drawing demonstrates the deforming forces on the proximal humerus in the setting of fracture. The supraspinatus (A) exerts a force posteromedially. The infraspinatus and teres minor (B) pull posteromedially and externally rotate. The subscapularis (C) exerts an anteromedially directed force on the lesser tuberosity. The pectoralis major (D) internally rotates and adducts, while the deltoid (E) pulls superiorly on the metadiaphysis of the humerus. (Reprinted with permission from Gruson et al. [4]).

When fractured, the greater and lesser tuberosities are deformed by their musculotendinous rotator cuff attachments (Figure 1) [2, 3].

**Figure 2:**
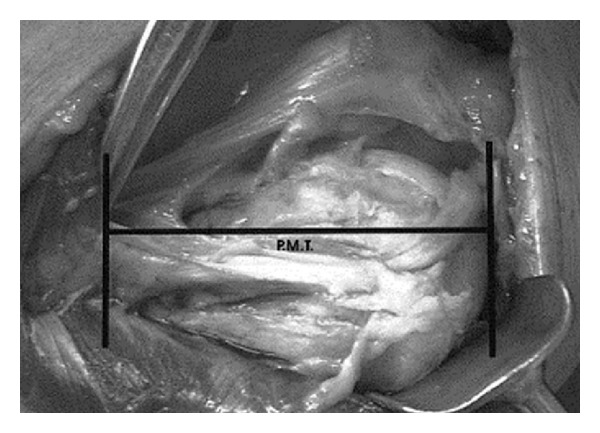
The average distance from the pectoralis major tendon (PMT) insertion to the tangent to the humeral head is 5.6 cm. (Reprinted with permission from Murachovsky et al. [5]).

The supraspinatus muscle, innervated by the suprascapular nerve, attaches to the superior facet of the greater tuberosity with a force vector that pulls predominantly in a medial direction. The infraspinatus muscle, also innervated by the suprascapular nerve, inserts on the middle facet of the greater tuberosity. The teres minor muscle, innervated by the axillary nerve, attaches to the inferior facet. Together, these three externally rotate and yield a posteromedially directed deforming force. Therefore, if the greater tuberosity is fractured, it is displaced posteromedially. If it remains intact, and there is a surgical neck fracture, the resulting deformity is typically varus and external rotation. Anteriorly, the subscapularis, innervated by the upper and lower subscapular nerves, attaches to the lesser tuberosity, resulting in anteromedial displacement of this osseous fragment if fractured [2, 3]. The pectoralis major tendon insertion is an important landmark, especially during hemiarthroplasty. Murachovsky et al. showed that the average distance from the pectoralis major tendon insertion to the tangent to the humeral head was 5.6 cm (Figure 2) [5].

Torrens and colleagues confirmed this relationship and added that hemiarthroplasty rotation could also be estimated based on the insertion of this tendon [6]. Specifically, the authors found that the anatomy of the proximal humerus can be restored by placing the prosthesis 5.6 cm above the upper insertion of the pectoralis major [6]. Additionally, the authors found the distance from the upper margin of the pectoralis major insertion to be 17.55% of the total humeral length. Thus, for a more anatomic reconstruction, the authors suggest acquiring a preoperative radiograph of the contralateral humerus and calculating the patient-specific length based on measurements made off of the unaffected side [6].

Humeral head vascularity comes from the anterior and posterior humeral circumflex arteries. The anterior humeral circumflex artery (AHCA) runs with its two venae comunicantes as the “three sisters” before anastomosing with the posterior humeral circumflex artery (PHCA). The AHCA gives off an anterolateral ascending branch that crosses the subscapularis tendon anteriorly and runs superiorly along the lateral border of the intertubercular groove before terminating as the arcuate artery [2, 3].

The PHCA runs posteriorly along with the axillary nerve through the quadrangular space, defined by the subscapularis and teres minor muscles superiorly, the teres major inferiorly, the long head of the triceps medially, and the humeral surgical neck laterally [2]. Within the quadrangular space, the PHCA splits into posterior and anterior branches, which run in concert with the branches of the axillary nerve to supply the proximal humerus and deltoid muscle.

**Figure 3:**
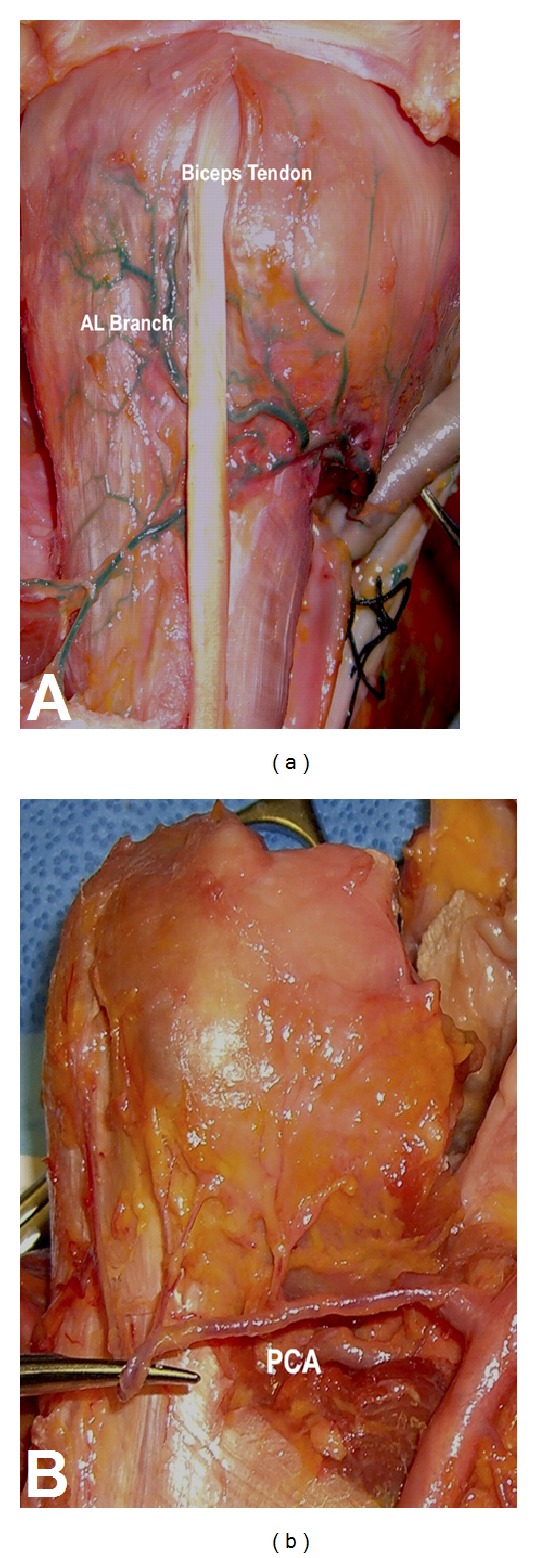
The anterior humeral circumflex is seen adherent to the proximal humerus (a), whereas the posterior humeral circumflex artery (PCA) seen in (b) is less adherent with several perforating branches along its course, making this vessel less vulnerable to injury in the case of proximal humerus fracture. (Reprinted with permission from Hettrich [10]).

Classically, the AHCA has been considered the most important blood supply to the proximal humerus, with secondary contributions from the PHCA and muscular attachments of the proximal humerus [3, 7–9]. More recently, however, data suggests that the contribution of the PHCA is more substantial than previously believed. Hettrich et al. demonstrated that the majority of the blood supply to the proximal humerus actually arises from the PHCA [10]. Specifically, the authors report that 64% of humeral head perfusion arises from the PHCA, while the AHCA only contributed 36% of humeral head perfusion. Therefore, in the setting of proximal humerus fracture, such results indicate that damage to the humeral head blood supply may be preserved, even in cases where the AHCA is disrupted (Figure 3).

**Figure 4:**
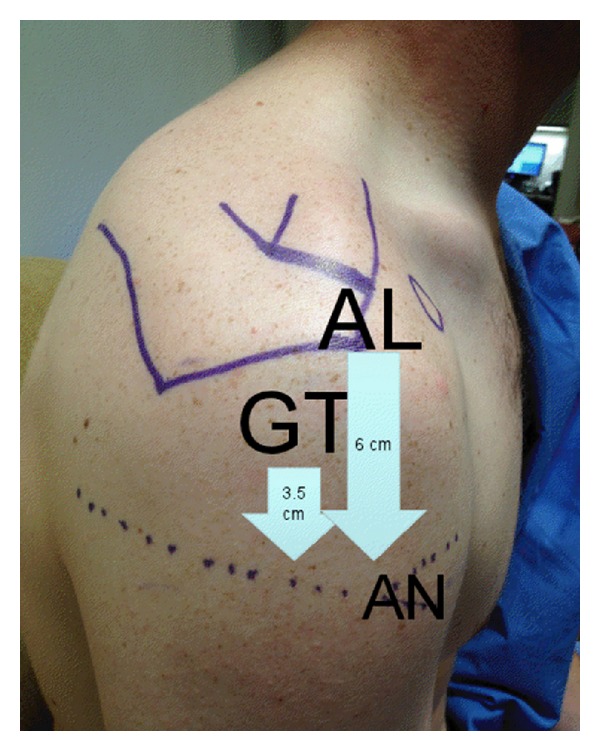
The axillary neurovascular structure (AN) can be palpated as a cordlike structure within the anterior deltoid raphe located an average of 3.5 cm from the greater tuberosity (GT) prominence or 6 cm from the anterolateral (AL) border of the acromion, the latter of which is more reliable in the setting of proximal humerus fracture.

The axillary nerve is the most frequently injured nerve in proximal humerus fractures, and the suprascapular nerve is the second most commonly injured [11]. The axillary nerve enters the quadrangular space along with the PCHA at an average distance of 1.7 cm from the surgical neck [2]. In the quadrangular space, the nerve divides into anterior and posterior branches, the latter of which provides motor input to the posterior and middle heads of the deltoid before terminating as the superior lateral brachial cutaneous nerve [2]. The anterior branch of the axillary nerve continues along the undersurface of the deltoid, crosses the anterior deltoid raphe, the avascular region separating the anterior and middle heads of the deltoid, at an average of 3.5 cm from the lateral prominence of the greater tuberosity and 6.3 cm from the anterolateral border of the acromion (Figure 4) [12]. Gardner and colleagues showed that this nerve can be reliably palpated as a cord-like structure at this location. The proximity of the axillary nerve makes it particularly vulnerable to both traumatic and iatrogenic injury [13].

The suprascapular nerve runs posteroinferiorly through the suprascapular notch to supply the supraspinatus and infraspinatus muscles. The motor branch of the nerve arises about 1 cm from the base of the scapular spine and courses 2 mm posterior to the glenoid rim [2]. The nerve is particularly vulnerable to traction injury at two distinct locations: its branch-point from the upper trunk and at the suprascapular notch where it runs deep to the transverse scapular ligament.

## 3. Clinical Evaluation

### 3.1. History and Physical

Evaluation of a proximal humerus fracture begins with a thorough history and physical examination. While one may focus attention on the shoulder, it is important to consider associated injuries and concomitant pathology of the shoulder girdle, upper extremity, and cervical spine. The most common mechanism is a fall from standing in an elderly, osteoporotic woman [14]. Other, less common causes, include high-energy traumas such as falls from height, motor vehicle crashes, seizures, and electric shock. It is possible to have concomitant glenohumeral dislocation [15]. Recognition of a proximal humerus fracture dislocation is imperative. Pathologic proximal humerus fractures can be seen in the setting of neoplastic or metabolic bone disease. Such consideration is especially important in young patients with this injury and a low energy mechanism.

The patient's baseline level of function, hand dominance, functional demand, and ability to participate in rehabilitation must be assessed as these factors contribute to clinical management decisions. Patients with proximal humerus fractures commonly present with a swollen, ecchymotic shoulder with pain and limited range of motion. The skin should be inspected for associated lesions, ecchymosis, and prior surgical scars. Gross deformity may indicate glenohumeral dislocation. A diligent neurovascular exam is crucial, with particular attention paid to axillary nerve function. Although rare, acute neurovascular compromise may indicate the need for emergent surgical intervention, especially in high-energy injures [16]. Sluggish capillary refill, weak distal pulses, and hypoesthesias are all warning signs of vascular compromise.

**Figure 5:**
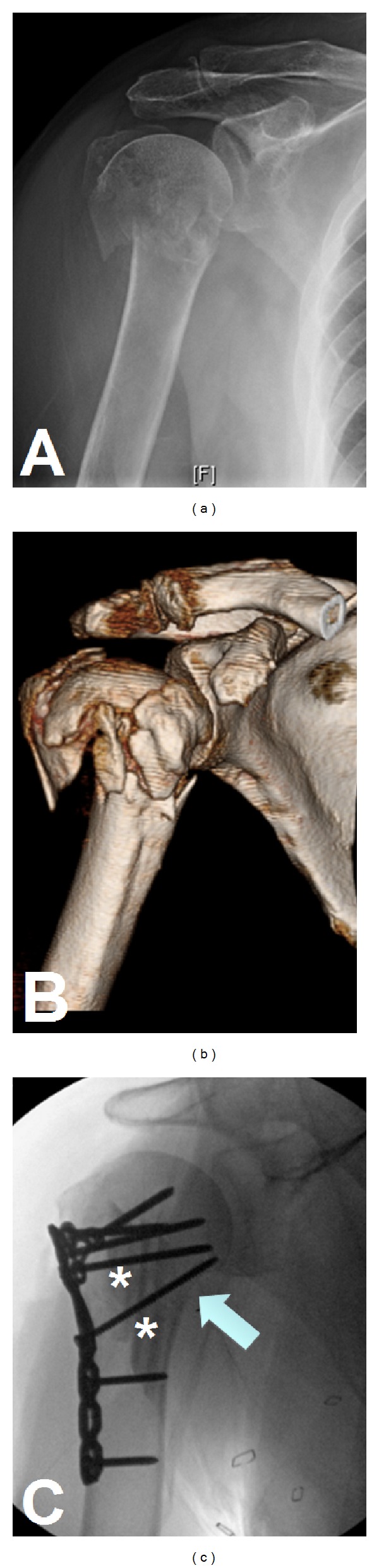
Radiograph (a) of a proximal humerus fracture. The CT scan with 3D reconstruction (b) adds significant detail and aids in preoperative planning. Osteosynthesis was carried out (c) with the use of intramedullary fibulas (asterisks) and particular attention paid to restoration of the medial calcar (arrow).

The trauma series of the shoulder consists of three set views: true AP, the lateral or scapular-Y, and axillary views. These allow for adequate evaluation of fracture anatomy, comminution, and fragment displacement. The true AP radiograph is taken perpendicular to the glenohumeral joint by angling the beam 40 degrees away from midline to account for scapulothoracic and glenoid version. The axillary view provides an accurate view of the glenohumeral relationship and is critical to confirm location of the humeral head. Alternatively, in patients who are unable to tolerate this view secondary to pain, a Valpeau view may be substituted. Computed tomography (CT) is a very useful imaging modality. It is helpful for surgical planning as it allows improved delineation of fracture displacement, assessment of comminution, and evaluation of the articular surface [16]. We routinely obtain CT scans with 3D reconstruction views in all operative candidates (Figures 5(a) and 5(b)). Magnetic resonance imaging (MRI) is rarely indicated in the acute setting but becomes a useful tool if underlying bony pathology or malignancy is suspected.

### 3.2. Classification

The AO classification for proximal humerus fractures was initially described by Muller in 1988 and divides the fracture patterns into the classic 27 subgroups based on the location, type, and severity of the fracture [17]. This scheme is rarely used in the clinical setting. Numerous other classifications have been described, including the Kocher, Codman, and Jakob and Ganz systems. The most widely employed, however, is the Neer classification. Neer divides the proximal humerus into 4 conceptual and functional “parts”: the greater tuberosity, the lesser tuberosity, the articular segment (head), and the humeral shaft [18]. In order to qualify as a part, the fragment must have greater than 1 cm of displacement or 45 degrees of angulation. The greater tuberosity is an exception to this rule, requiring only 0.5 cm of displacement to be considered a part [18].

## 4. Treatment Options

### 4.1. Nonoperative Treatment

Nondisplaced and minimally displaced fractures may be treated conservatively. Following two weeks of sling immobilization, passive motion of the shoulder commences. Shorter periods of immobilization are associated with lower pain scores in the short term; however, at 6 months, there was no difference [19]. More recently, Lefevre-Colau and colleagues found that early mobilization for impacted proximal humerus fractures was safe and more effective for performance restoration than a period of immobilization as traditional teaching would suggest [20]. Patients should be followed with serial radiographs to evaluate for fracture displacement. We recommend radiographs at two weeks (prior to initiation of motion) and then again at 3 weeks to ensure fracture stability.

The literature supports good to excellent outcomes for nonoperative management of these minimally displaced fractures in the elderly population. Particularly with regard to valgus impacted fracture patterns, 80% of patients report good to excellent outcomes. Further, patients regained approximately 80% of the abduction and flexion strength compared with the contralateral extremity [21]. Court-Brown et al. reported similarly positive results in displaced 2-part translated fractures treated nonoperatively, noting that surgery did not improve outcome in elderly patients when compared to nonoperative management regardless of the degree of initial translation [22].

In a subset of patients, elderly, low demand, or those with significant medical comorbidities, even more complex fractures patterns may be treated without surgery. Outcomes in nonoperatively managed 3- and 4-part fractures found that age was the most significant factor influencing outcomes, and no significant correlation was found based on fracture pattern or even radiographic outcomes after union [23].

### 4.2. Percutaneous Pinning

Percutaneous pinning is a minimally invasive technique with limited indications. Amenable fracture patterns include 2-part proximal humerus fractures, ideally of the surgical neck, and 3- or 4-part fractures with adequate bone stock [24]. Theoretically, this technique limits iatrogenic vascular compromise, postoperative pain, operative time, and blood loss while improving cosmesis. Good outcomes can be achieved 70% of the time in 2-part fracture patterns [25]. Comparison of percutaneous techniques in all fracture patterns revealed, as one may expect, that 4-part fractures had the poorest results [26].

Better outcomes are reported using percutaneous fixation in patients with good bone quality, an intact medial calcar, lack of proximal shaft comminution, and stable fixation under dynamic fluoroscopy [27]. Reported complications of this technique include pin track infections, avascular necrosis of the humeral head, and pin migration with resultant loss of reduction [24]. Longer term followup of patients treated with percutaneous fixation revealed greater prevalence of osteonecrosis and posttraumatic osteoarthritis than previously reported [28].

### 4.3. Intramedullary Fixation

Intramedullary devices can be used for fixation of 2-, 3-, and 4-part fractures. Most successful outcomes occur in 2-part fractures. Intramedullary nail fixation with indirect reduction has the benefit of decreased soft tissue stripping. A retrospective review of 2-, 3-, and 4-part fractures treated with a Polarus nail reported that lateral metaphyseal comminution and a lateralized starting point into the greater tuberosity increased the risk of fixation failure [29]. These authors reported a very high complication rate in their series with only eleven of the twenty fractures healing without complications. Another study reported a 45% reoperation rate in 3- and 4-part proximal humerus fractures treated with the Polarus nail [30]. A similar cohort of patients treated with the Telegraph nail reported the best outcomes in those with extraarticular surgical neck fractures. Similar to plate fixation, complications of intramedullary nailing include screw penetration, nail impingement, hardware migration, and failure of fixation. More recently, Lobenhoffer and Mathews [31] reported an average Constant score of 60.0 in 99 proximal humerus fractures treated surgically with the Targon nail at 7 months postoperatively.

At our institution, intramedullary nailing of proximal humerus fractures is rarely indicated. However, Konrad et al. have shown equivalent outcomes with plate compared with nail fixation of three-part proximal humerus fractures [32]. Similarly, a prospective randomized trial of plate versus nail for two-part surgical neck fractures also found no difference in clinical outcomes scores at three-year followup [33]. These data suggest that intramedullary devices continue to have a role in the management of certain proximal humerus fractures.

### 4.4. ORIF

Osteosynthesis is indicated for 2-, 3-, and 4- part fractures in appropriate patients. Exceptions include some 4-part fractures, head-splitting fractures, and fracture-dislocations, which are indicated for prosthetic replacement. While plate fixation has been shown to have superior patient outcome scores when compared with nonoperative treatment in elderly patients, a recent randomized controlled trial showed better radiographic outcomes for plate fixation but equivalent functional outcomes in three- and four-part fractures [34, 35]. Classically, indications for fixation in 4-part fractures include valgus impaction with preservation of the medial capsular blood supply [36]. In our experience, however, more complex 4-part patterns can successfully be treated with ORIF. Complications with osteosynthesis, however, remain high. Particularly in patients with osteopenic or osteoporotic bone, high rates of intraarticular screw penetration have been reported [37]. This can lead to subsequent impingement from plate migration, nonunion, malunion, or intraarticular penetration of screws [38–40]. In [40] the risk of avascular necrosis (AVN) secondary to vascular compromise is greater in more complex fracture patterns and may be compounded by iatrogenic soft tissue stripping. While this concern still exists, the correlation between head perfusion and development of ischemia is more complex than initially thought. Hertel et al. initially observed that predictors of humeral head ischemia as based on intraosseous laser Doppler flowmetry were metaphyseal head extension, integrity of medial hinge, and basic fracture pattern [41]. These patients were followed long term, and it was found that in fractures demonstrating intraoperative ischemia, 8/10 did not go on to humeral head collapse from AVN, and the other 2/10 demonstrated collapse at mean 5 year followup. In those fractures without intraoperative ischemia, 4/30 still went on to humeral head collapse from AVN. Clearly, humeral head ischemia is not the only factor leading to AVN in proximal humerus fracture as most fractures with intraoperative ischemia did not go on to collapse [42].

Indications for open reduction internal fixation techniques of proximal humerus fractures have expanded with the introduction of locking plate technology. Initial data on specific locked fixation devices such as the PHILOS plate nearly eliminated complications due to hardware failure and subacromial impingement with good functional outcomes if correct surgical technique is employed. Constant scores in the long term for patients fixed with locked plates range from 70 to 76 [43–45]. Sudkamp et al. found poorer Constant scores in female patients over 40 years of age with varus deformity greater than 20 degrees [46]. A delay in the initiation of rehabilitation due to medical comorbidities has also been found to lead to poor outcomes [47]. Initial fracture pattern with varus extension angulation has also been found to do poorly when compared with valgus impacted angulation pattern [48].

The majority of complications seen in locked plating are related to surgical technique. The surgeon must therefore restore the medial calcar, avoid a varus malreduction, and prevent screw penetration to ensure best outcomes, decreasing revision rates and loss of fixation [38, 46, 49–51]. Techniques to avoid these complications include use of fibular strut allograft as a medial endosteal implant to prevent varus collapse [52]. Screw depth sounding has also been described to avoid intraarticular screw penetration [53].

Optimal management of 4-part proximal humerus fractures is most controversial. A systematic review of the available evidence-based literature was inconclusive with regard to arthroplasty versus internal fixation for these fractures [54]. In our experience, however, younger, high-demand patients almost always benefit from restoration of their native anatomy. One of our senior authors (D. G. Lorich) has championed the use of an intramedullary fibula strut graft (Figure 5(c)), which he uses as both a reduction aid and as a structural augment for screw purchase in patients with poor bone quality. Patients treated with this fixation method did not have any intraarticular screw penetration or hardware cut-out and had high outcome scores [55]. Ultimately, the decision to pursue ORIF in 4-part proximal humerus is based upon patient specific factors and the technical ability of the surgeon.

### 4.5. Arthroplasty

Arthroplasty for proximal humerus fractures is a good surgical option for low-demand elderly patients, or fractures that are not amenable to ORIF. Significant controversy exists as to the best surgical intervention for 3- or 4-part fractures in the elderly osteoporotic patient. As previously noted, 4-part valgus impacted fractures have a lower rate of osteonecrosis due to preservation of blood supply and may be more amenable to fixation compared with displaced 4-part fractures [56, 57]. The current literature suggests that a good candidate for hemiarthroplasty is the elderly low-demand patient in whom anatomic reduction cannot be achieved with internal fixation [57]. These patients have been shown to have significantly less pain after hemiarthroplasty compared with nonoperative treatment, although range of motion is similar [58]. Patients who present with initial varus angulation greater than 30 degrees are at increased risk for fixation failure, and thus hemiarthroplasty may decrease their need for reoperation [46]. Further, fracture-dislocations may do poorly following osteosynthesis and thus should be treated with arthroplasty except in the young patient.

It is also important to consider the degree of underlying shoulder pathology, including symptomatic glenohumeral osteoarthritis, or rotator cuff arthropathy. If present, these could potentially predispose a patient to poor outcomes following osteosynthesis. Thus, the presence of osteoarthritis or rotator cuff pathology should influence the surgeon's choice away from ORIF and towards arthroplasty (either hemiarthroplasty or reverse total shoulder arthroplasty).

Anatomic tuberosity healing enables a functional rotator cuff and is critical for patient satisfaction and functional outcomes following hemiarthroplasty. Boileau et al. [59] found that tuberosity malpositioning occurred in half of patients who underwent hemiarthroplasty for proximal humerus fracture. This was also highly correlated with unsatisfactory results, prosthesis malalignment, decreased range of motion, and residual pain. Tuberosity healing and outcomes may be improved by use of a fracture specific humeral component (79%) compared with those treated by a conventional stem (66%).

There is debate in the literature as to hemiarthroplasty versus reverse total shoulder arthroplasty for acute proximal humerus fractures. Currently, indications for a reverse total shoulder arthroplasty in proximal humerus fracture are limited to rotator cuff deficiency and severe tuberosity comminution. Recently, outcomes data comparing hemiarthroplasty with reverse total shoulder arthroplasty for acute proximal humerus fractures showed superior function in patients who underwent reverse total shoulder arthroplasty [60, 61]. In a patient where there is concern for tuberosity healing due to tuberosity comminution, a reverse shoulder arthroplasty avoids reliance on the rotator cuff and provides the patient with a functional shoulder. While prospective data is needed, indications for reverse total shoulder arthroplasty may expand.

Another important consideration is timing to intervention. Arthroplasty within the first 4 weeks following injury yields superior functional results when compared with delayed procedures or procedures for malunion [62, 63]. In the patients with glenohumeral arthritis and risk of tuberosity nonunion, a reverse total shoulder replacement may yield better functional outcomes.

## 5. Authors' Preferred Treatment Algorithm

**Figure 6:**
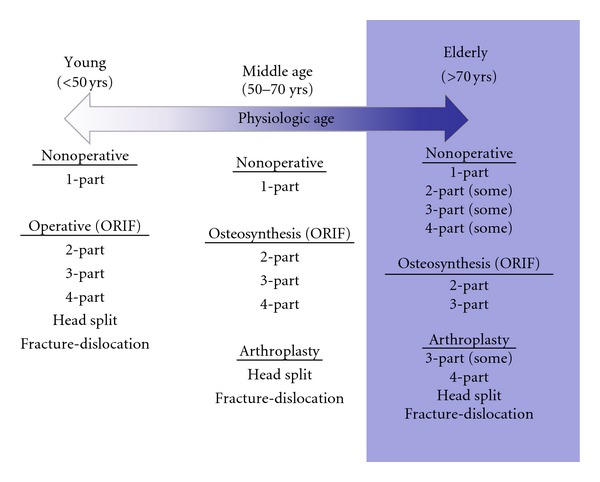
Treatment algorithm for proximal humerus is based on patients chronological and physiological age. Young patients are treated more aggressively with osteosynthesis making every attempt to restore normal anatomy, while the older patient may benefit from an array of treatments ranging from nonoperative to prosthetic replacement.

As discussed ealier, intervention ranges from nonoperative treatment to osteosynthesis and arthroplasty. Our treatment algorithm is based upon both chronological age as well as physiologic age (Figure 6). We divide patients into three groups—the young, the middle aged, and the elderly.

Patients under the age of 50 should receive every effort to restore normal anatomy including those high-risk fracture patterns such as 4-part and some head splits or fracture-dislocations. Elderly patients are those over 70 years of age. These patients benefit from osteosynthesis of 2- and 3-part and some 4-part proximal humerus fractures. The majority of 4-part fractures, head splits, and fracture-dislocations should be treated with prosthetic replacement in this group. Patients aged 50 to 70 years represent a gray area. That is, patients who are physiologically young may be treated more aggressively with osteosynthesis. Conversely, the physiologically elderly should be treated as such.

**Figure 7:**
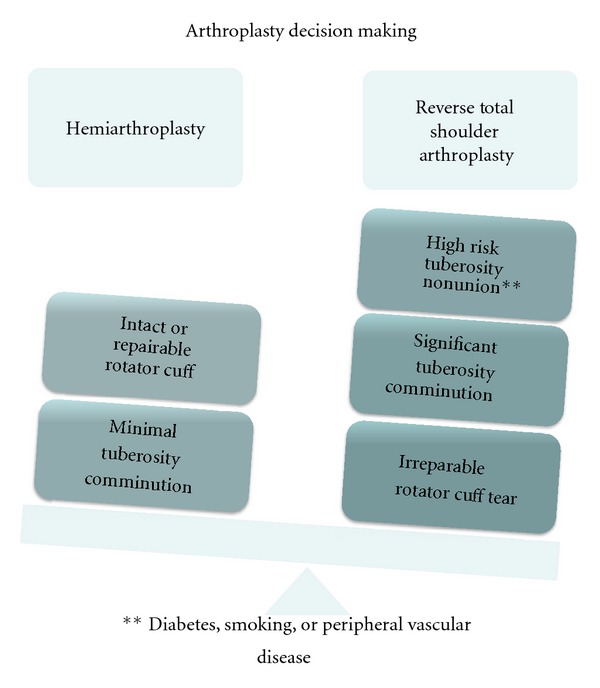
In patients indicated for prosthetic replacement, it is important to consider rotator cuff competency and risk of tuberosity nonunion. Patient with high risk of tuberosity nonunion (severe comminution, diabetes, smoking, or peripheral vascular disease) may benefit from primary reverse total shoulder arthroplasty.

If arthroplasty is to be employed, the decision between hemiarthroplasty and reverse total shoulder arthroplasty is based upon several factors (Figure 7).

Hemiarthroplasty requires an intact rotator cuff (or repairable rotator cuff) and tuberosities with a high likelihood of healing. Reverse total shoulder arthroplasty should be considered in patients with an irreparable rotator cuff, comminuted tuberosities, and those with comorbidities (diabetes, smoking, or peripheral vascular disease) that put them at risk for tuberosity nonunion.

## 6. Summary

Proximal humerus fractures are common injuries, especially among older osteoporotic women. Restoration of function requires a thorough understanding of the neurovascular, musculotendinous, and bony anatomy. In the vast majority of cases, proximal humerus fractures may be treated nonoperatively. In displaced fractures, however, surgical intervention may be pursued. While numerous constructs have been investigated, the proximal humerus locking plate is most widely used and effective. In our experience, with proper restoration of the medial calcar, even 3- and 4-part proximal humerus fractures may be effectively treated with ORIF. Arthroplasty is reserved for fractures that cannot be reconstructed, such as comminuted 4-part fractures, head-split fractures, or fractures with significant underlying arthritic changes. Reverse total shoulder arthroplasty is reserved for patients with a deficient rotator cuff, or highly comminuted tuberosities.
